# The Cytoprotective Effects of *E*-α-(4-Methoxyphenyl)-2’,3,4,4'-Tetramethoxychalcone (*E*-α-*p*-OMe-C_6_H_4_-TMC)—A Novel and Non-Cytotoxic HO-1 Inducer

**DOI:** 10.1371/journal.pone.0142932

**Published:** 2015-11-13

**Authors:** Kai B. Kaufmann, Nafisah Al-Rifai, Felix Ulbrich, Nils Schallner, Hannelore Rücker, Monika Enzinger, Hermina Petkes, Sebastian Pitzl, Ulrich Goebel, Sabine Amslinger

**Affiliations:** 1 Department of Anesthesiology and Intensive Care Medicine, University Medical Center Freiburg, Freiburg, Germany; 2 Institute of Organic Chemistry, University of Regensburg, Regensburg, Germany; 3 Institute of Pharmaceutical Biology, University of Regensburg, Regensburg, Germany; University of Catania, ITALY

## Abstract

Cell protection against different noxious stimuli like oxidative stress or chemical toxins plays a central role in the treatment of many diseases. The inducible heme oxygenase isoform, heme oxygenase-1 (HO-1), is known to protect cells against a variety of harmful conditions including apoptosis. Because a number of medium strong electrophiles from a series of α-X-substituted 2’,3,4,4’-tetramethoxychalcones (α-X-TMCs, X = H, F, Cl, Br, I, CN, Me, *p*-NO_2_-C_6_H_4_, Ph, *p*-OMe-C_6_H_4_, NO_2_, CF_3_, COOEt, COOH) had proven to activate Nrf2 resulting in HO-1 induction and inhibit NF-κB downstream target genes, their protective effect against staurosporine induced apoptosis and reactive oxygen species (ROS) production was investigated. RAW264.7 macrophages treated with 19 different chalcones (15 α-X-TMCs, chalcone, 2’-hydroxychalcone, calythropsin and 2’-hydroxy-3,4,4’-trimethoxychalcone) prior to staurosporine treatment were analyzed for apoptosis and ROS production, as well as HO-1 protein expression and enzyme activity. Additionally, Nrf2 and NF-κB activity was assessed. We found that amongst all tested chalcones only *E*-α-(4-methoxyphenyl)-2’,3,4,4'-tetramethoxychalcone (*E*-α-*p*-OMe-C_6_H_4_-TMC) demonstrated a distinct, statistically significant antiapoptotic effect in a dose dependent manner, showing no toxic effects, while its double bond isomer *Z*-α-*p*-OMe-C_6_H_4_-TMC displayed no significant activity. Also, *E*-α-*p*-OMe-C_6_H_4_-TMC induced HO-1 protein expression and increased HO-1 activity, whilst inhibition of HO-1 by SnPP-IX abolished its antiapoptotic effect. The only weakly electrophilic chalcone *E*-α-*p*-OMe-C_6_H_4_-TMC reduced the staurosporine triggered formation of ROS, while inducing the translocation of Nrf2 into the nucleus. Furthermore, staurosporine induced NF-κB activity was attenuated following *E*-α-*p*-OMe-C_6_H_4_-TMC treatment. Overall, *E*-α-*p*-OMe-C_6_H_4_-TMC demonstrated its effective cytoprotective potential via a non-toxic induction of HO-1 in RAW264.7 macrophages. The observed cytoprotective effect may partly be related to both, the activation of the Nrf2- and inhibition of the NF-κB pathway.

## Introduction

While heme oxygenase-2 (HO-2) is constitutively expressed in most tissues, the inducible isoform of heme oxygenases HO-1 represents a powerful response to a variety of adverse stimuli including ischemia-reperfusion injury, hypoxia or toxicity, thus leading to cellular protection [1]. These cytoprotective effects of HO-1 are caused by various factors. Firstly, HO-1 eliminates free heme which would otherwise lead to apoptosis in increased concentrations [2, 3]. Secondly, the cytoprotective function of HO-1 is attributed to its enzymatic reaction products: biliverdin, which is further transformed to bilirubin by biliverdin reductase, iron and carbon monoxide [4, 5]. In general, cytoprotection involves the inhibition of apoptosis and its related pathways. More and more results indicate that HO-1 induction is a promising therapeutic regime to treat a variety of diseases [1, 6]. Apart from that, HO-1 may play an important role in sepsis, where patients suffer from immune suppression as a result of an increased amount of apoptotic immune cells [7, 8].

The pharmacological application of carbon monoxide or biliverdin/bilirubin can at least partly replicate HO-1 dependent cytoprotective effects. Low concentrations of inhaled carbon monoxide can produce anti-inflammatory and antiapoptotic effects, but *in-vivo* applications of inhaled carbon monoxide are limited due to its considerable toxicity [5, 9–11]. Therefore carbon monoxide releasing molecules (CO-RMs) are considered as attractive alternatives [12–14]. However, there are some disadvantages when looking at the intravenous application of CO-RMs in humans. After the release of CO, additional degradation products of the CO-RMs appear which might be toxic. Additionally, depending on the CO release mechanism such as medium-induced hydrolytic cleavage [13], application of light [15] or the action of cellular proteolytic enzymes [16, 17] different CO forming efficacies and kinetics must be suspected. Thus, the application of defined doses of CO remains challenging.

Since it is unclear whether the single end products of the HO-1 reaction exert potent therapeutic properties or if HO-1 activity itself contributes to the broad range of cytoprotective effects, we were interested in developing non-cytotoxic HO-1 inducers.

Various structurally different natural products exhibit cytoprotective effects by HO-1 induction. Amongst them are many examples with an α,β-unsaturated carbonyl unit that can act as an electrophilic Michael acceptor functionality alkylating reactive cysteine residues. Thereby, thiol-dependent signaling pathways like the Keap1-Nrf2 or NF-κB pathway can be addressed assuming an underlying covalent binding mode of action. HO-1 expression is in part regulated by the transcription factor Nrf2 which interacts with the antioxidant and electrophile response element (ARE/EpRE) [18]. While Nrf2 is a key player in cytoprotection, NF-κB is one of the main inflammation-related transcription factors.

In a recent screening study using mostly natural products we found that all tested chalcones (1,3-diphenylprop-2-enones) gave a 2–6 fold induction of HO-1 activity in RAW264.7 cells [19]. Moreover, we could show that a chemical characterization of natural and synthetic chalcones by a kinetic thiol reactivity assay could be translated into biological activities such as HO-1 induction and inhibition of proinflammatory proteins such as iNOS and TNF [20–22], but also STAT5 inhibition [23]. The chalcones we used were mainly α-X-substituted 2’,3,4,4’-tetramethoxychalcones (α-X-TMCs, X = H, F, Cl, Br, I, CN, Me, *p*-NO_2_-C_6_H_4_, Ph, *p*-OMe-C_6_H_4_, NO_2_, CF_3_, COOEt, COOH) whose electrophilic behavior could be fine-tuned by the introduction of the extra substituent X in the α-position of the α,β-unsaturated carbonyl system [21]. Despite the fact that clearly electrophilic α-X-TMCs showed the best activities [22] suggesting a covalent mode of action, some (*E*)- and (*Z*)-1,2,3-triarylprop-2-enones (corresponding to α-Ar-chalcones) recently proved to be potent selective COX-2 inhibitors [24] as well as microtubule polymerization inhibitors [25] indicating a noncovalent binding mode. Since chalcones possess a high structural variability with the two *E*/*Z* double bond isomers, their conformational freedom (including *s*-cis vs. *s*-trans form) as well as quite different electronic properties which depend on additional substituents, the mechanisms by which they exert their antiapoptotic effect are not well understood.

We demonstrate that out of a library of 15 electrophilicity-tuned α-X-chalcones (α-X-TMCs) and 4 further chalcones (chalcone, 2’-hydroxychalcone, calythropsin, 2’-hydroxy-3,4,4’-trimethoxychalcone (α-H-HC)) the very weak electrophile *E*-α-*p*-OMe-C_6_H_4_-TMC exerts an effective cytoprotective potential by non-cytotoxic HO-1 induction in RAW264.7 macrophages via the Nrf2 pathway.

## Materials and Methods

### Synthesis of chalcone derivatives

α-X-TMCs and other chalcones were prepared as described previously [20, 21]. *Z*-α-*p*-OMe-C_6_H_4_-TMC was isolated from the *E/Z*-isomeric mixture of ~ 80:20 obtained by the same method as described before [21] in a yield of 15% by column chromatography on silica gel and preparative TLC using petroleum ether-EtOAc mixtures as the eluents (analytical data see supporting information). Other compounds were purchased from the following commercial sources and used without further purification: hemin and gelatin (from cold water fish skin) from Sigma-Aldrich (Taufkirchen, Germany), NADPH from AppliChem (Darmstadt, Germany), OPD (ortho-phenylenediamine dihydrochloride) from Acros Organics (Geel, Belgium), bilirubin from Frontier Scientific (UK), Triton X-100 from Merck (Darmstadt, Germany).

### Cell culture and experimental protocol

RAW264.7 macrophages (ATCC No. TIB-71) were cultured in RPMI 1640 medium supplemented with 10% fetal calf serum, 2 mM glutamine and 1% penicillin and streptomycin at a constant temperature of 37°C in humidified air containing 5% carbon dioxide. The cells were plated at least 48 h prior to the initiation of the experiment. Cells were grown to approximately 90% confluence in either 6-well cell culture plates (flow cytometry) or 10 cm cell culture dishes (protein analysis) under conditions described above. The chalcones were dissolved in DMSO under light protection (stock solution 10 mM) and were added immediately to the culture medium obtaining the indicated final concentration. Cells were pre-incubated with chalcones for 3 h and apoptosis was induced using staurosporine (1.0 μM for 2 h). For each experiment controls were obtained, keeping cells at standard conditions without intervention. Cells were harvested for analysis after the 2 h period of staurosporine incubation. For the inhibition of HO-1 protein expression and enzyme activity SnPP-IX (80 μM for 1 h) was added prior to incubation with chalcones and induction of apoptosis.

### Flow cytometry

Cells (approx. 1 × 10^5^) were washed in phosphate-buffered saline, trypsinized, resuspended in 100 μL binding buffer (0.010 M HEPES, 0.14 M NaCl, 2.5 mM CaCl_2_, pH 7.4) and stained with 5 μL FITC annexin-V and propidium iodide (PI). Samples were incubated at room temperature for 15 min before 400 μL of binding buffer was added. The percentage of annexin-V positive cells was measured using a flow cytometry device (FACS-Calibur®, Becton-Dickinson, Heidelberg, Germany). Fluorescence intensity of RAW264.7 macrophages was measured in fluorescence channel 1 (annexin-V) and in fluorescence channel 3 (propidium iodide). Unstained cells and those only stained with either annexin-V or propidium iodide served as negative controls for background fluorescence and for set up of compensation settings.

Reactive oxygen species were detected in RAW264.7 cells using the ROS detection kit (Enzo Life Sciences, Lörrach, Germany). Briefly, cells were harvested (3 × 10^5^), resuspended in 500 μL of ROS detection solution and stained for 30 min at 37°C in the dark. Samples were measured immediately using the flow cytometry device. The fluorescent product generated was analyzed in fluorescence channel 1.

### Western blotting

Nuclear and cytoplasmic protein fractions were obtained using a commercially available extraction kit (Nuclear Extract Kit, Active Motif Europe, Rixensart, Belgium). Equal amounts of cell extracts (30 μg) were boiled in 5 x SDS loading dye (50% glycerol, 0.50 M dithiothreitol, 350 mM SDS, 7.5 mM bromophenol blue, 250 mM TRIS, pH 6.8) for 5 min and subsequently subjected to 10% or 13% sodium-dodecyl-sulfate-polyacrylamide gel electrophoresis. Proteins were transferred onto PVDF membranes (Immobilon-P, Millipore, Schwalbach, Germany) using wet blotting technique after equilibrating the membranes in methanol (10 s), ddH_2_O (2 min) and wet blot buffer (20% methanol, 25 mM TRIS, 200 mM glycin, 5 min). After protein transfer, non-specific binding sites were blocked by incubating membranes in 5% milk powder dissolved in blocking solution (TBST; 10 mM TRIS, 150 mM NaCl, 0.20% Tween®, pH 8.0) for 1 h at room temperature. Subsequently, membranes were incubated with the respective primary antibody at the indicated concentration (HO-1 1:1000; Nrf2 1:1000) both from Cell Signaling (Danvers, MA, USA) overnight at 4°C. After incubation with a horseradish peroxidase-conjugated anti-rabbit secondary antibody (GE Healthcare, Freiburg, Germany, No. NA9340) proteins were visualized using the ECL plus Chemiluminescence Kit (GE Healthcare). For normalization, blots were reprobed with β-actin or lamin B1 both from Cell Signaling Technology (Danvers, MA, USA).

### HO-1 activity assay

The assay was performed as previously reported via *in-situ* formation of bilirubin by HO-1/BVR activity and quantification of bilirubin by ELISA [19]. Briefly, RAW264.7 macrophages (8 × 10^4^ cells) were placed in 96-well plates for 24 h and then treated with chalcones for 3 h, apoptosis was induced by staurosporine afterwards. Controls only received staurosporine to induce apoptosis and no chalcone treatment. After cell lysis (40 mM TRIS-HCl, pH 7.4, 250 mM sucrose, 137 mM NaCl, 10% (v/v) glycerin, 2.0 mM EDTA, 0.1% (v/v) Triton X-100, complete protease inhibitor cocktail, Roche Diagnostics, Germany) the HO-1 reaction mixture (40 mM TRIS-HCl, pH 7.4, 250 mM sucrose, 0.30 mM NADPH, 1.0 ng BVR (biliverdin reductase, Stressgen, Assay Designs, USA) and 2.5 μM hemin) was applied for 1 h. Bilirubin standards (0.50–2500 × 10^−9^ M bilirubin) were prepared in 40 mM TRIS-HCl, pH 7.4, 250 mM sucrose from a freshly prepared 10 mM stock solution in DMSO and combined with supernatant of whole cell lysates from control cells. Bilirubin was quantified by using an excess of the anti-bilirubin mouse-antibody 24G7 (Shino-Test, Japan, 0.57 μg μL^−1^ in 1% G-PBS with 0.50 mM sodium salicylate) and subsequently analyzing the unbound 24G7 by ELISA. To trap 24G7 an immunoplate (Nunc, Denmark) coated with a bilirubin-BSA conjugate (0.35 μg protein per well) was used. Detection was performed using a HRP-conjugated anti-mouse antibody from goat (Rockland, USA; 1:10000) with a freshly prepared substrate solution (0.40 g mL^−1^ OPD and 0.40 μL mL^−1^ 30% H_2_O_2_ in citrate buffer, pH 5.0). After quenching with aqueous 3.0 M H_2_SO_4_ the absorbance was measured at 492 nm (Multiskan Spectrum, Thermo, Finland). The sigmoidal calibration curve was fit to a four parameter logistic equation to determine unknown bilirubin concentrations. HO activity was calculated as pmol bilirubin formed per hour and per milligram of protein (pmol BR h^−1^mg^−1^) and assigned as HO-1 activity since small underlying HO-2 amounts should stay unchanged.

### NF-κB DNA binding activity assay

NF-κB DNA binding activity was analyzed using the TransAM™ NF-κB p65 method (Active Motif, Rixensart, Belgium), an ELISA-based kit detecting and quantifying the transcription factor activation. Nuclear protein fraction was obtained as mentioned above and analyzed according to the manufacturer’s instructions.

### Data analysis

Data was analyzed using a computerized statistical program (SigmaPlot Version 11.0, Systat Software Inc., San Jose, CA, USA). The Student’s *t* test or the Mann-Whitney *U* test (Wilcoxon rank-sum test) was used to determine whether a difference existed between two groups. P < 0.05 was considered statistically significant. The results are presented as means (± S.E.M.). When comparing more than two groups, a Kruskal–Wallis one-way analysis of variance (ANOVA) on Ranks or a one-way ANOVA was used with post-hoc Holm-Sidak test.

## Results

### The weak electrophile *E*-α-*p*-OMe-C_6_H_4_-TMC exerts antiapoptotic effects

**Fig 1:**
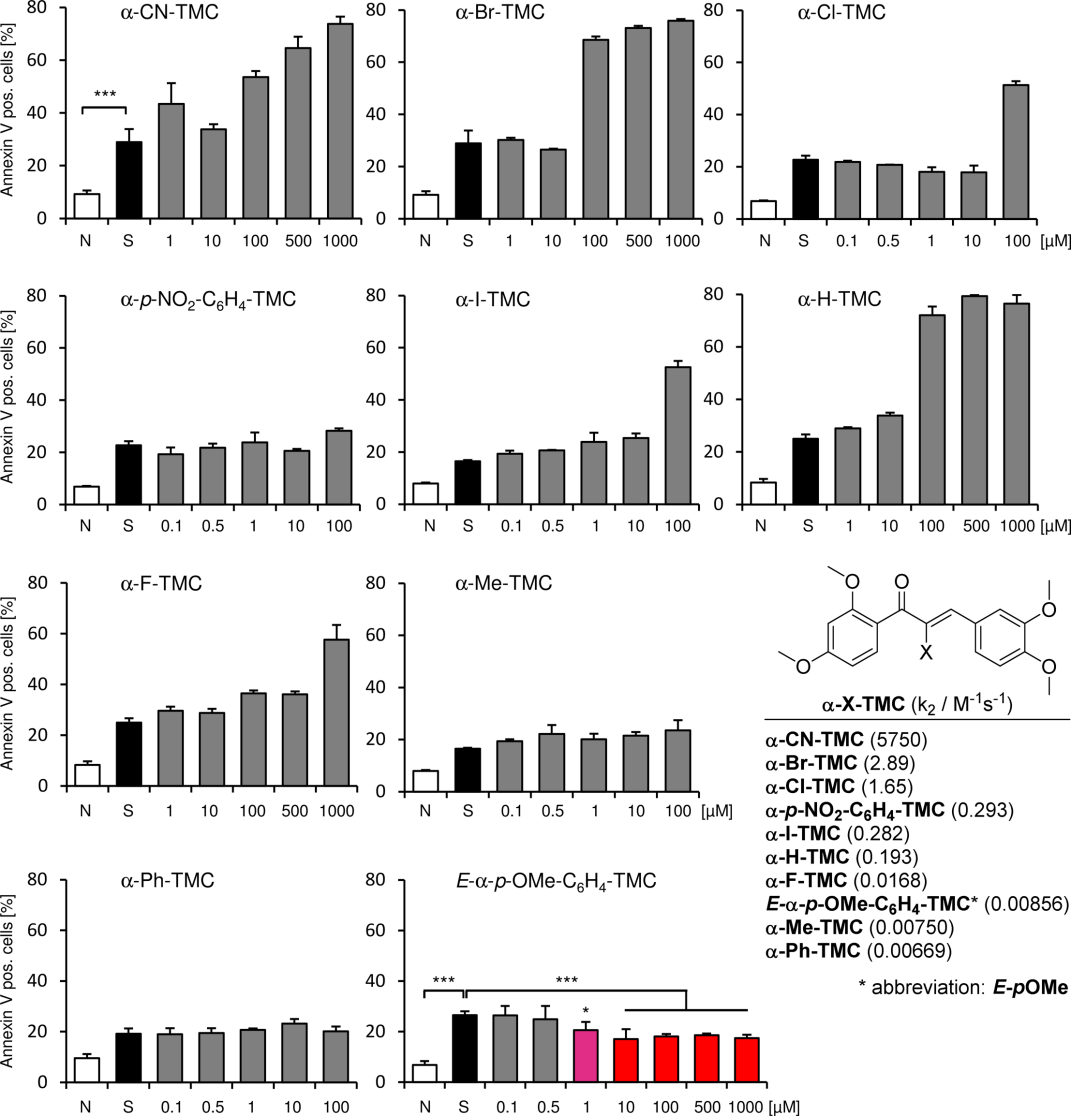
Flow cytometric analysis. Effect of different chalcones on staurosporine-induced apoptosis in RAW264.7 macrophages. Cells were pretreated with the indicated concentrations of chalcones for 3 h, before apoptosis was induced by staurosporine (1 μM for 2 h). Cells were harvested and stained with FITC annexin-V and propidium iodide. 1 × 10^4^ cells were analyzed in each experiment. (n = 4; mean ± S.E.M.; *** = p < 0.001 untreated vs. staurosporine and staurosporine vs. 10, 100, 500 and 1000 μM *E*-α-*p*-OMe-C_6_H_4_-TMC + staurosporine and * = p < 0.05 staurosporine vs. 1 μM *E*-α-*p*-OMe-C_6_H_4_-TMC + staurosporine). k_2_ values for the reaction with the model thiol cysteamine are taken from [21]. N, native; S, staurosporine.

**Fig 2:**
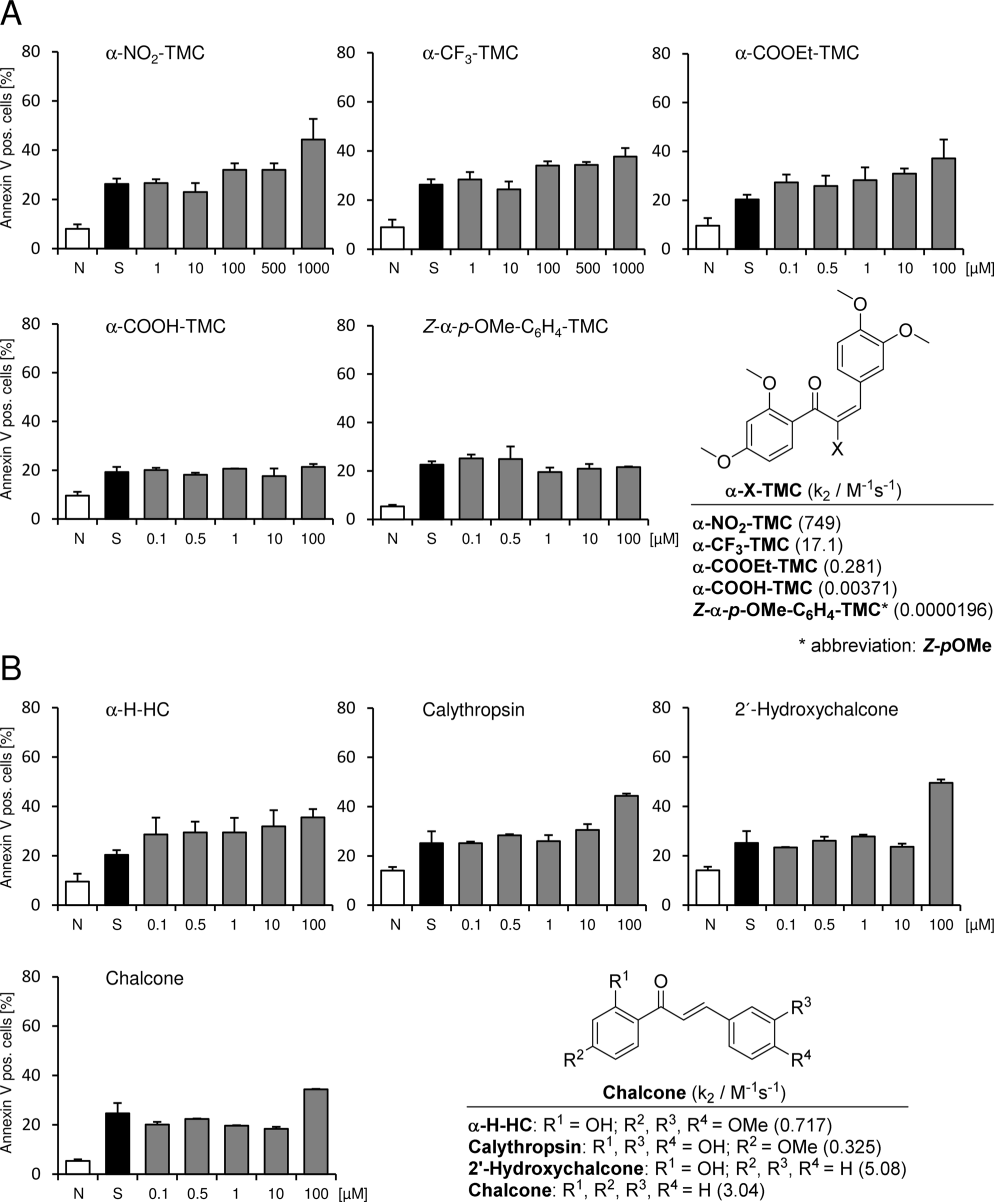
Flow cytometric analysis. Effect of different chalcones on staurosporine-induced apoptosis in RAW264.7 macrophages. Cells were pretreated with the indicated concentrations of chalcones for 3 h, apoptosis was induced by 1 μM staurosporine for 2 h afterwards. Cells were stained with FITC annexin-V and propidium iodide. 1 × 10^4^ cells were analyzed in each experiment. (n = 4; mean ± S.E.M.; *** = p < 0.001). k_2_ values for the reaction with the model thiol cysteamine are taken from [20, 21] and for *Z*-α-*p*-OMe-C_6_H_4_-TMC was determined in this study with 0.0000196 ± 0.0000021 M^-1^s^-1^. N, native; S, staurosporine.

We used our library of α-X-TMCs which possesses a wide range of electrophilicity together with the medium strong to moderate electrophilic chalcones 2’-hydroxychalcone, calythropsin, 2’-hydroxy-3,4,4’-trimethoxychalcone (α-H-HC) and chalcone itself. The second-order rate constants k_2_ for the reaction with the model thiol cysteamine are shown in Figs 1 and 2 [20, 21]. By applying these compounds we can utilize a chemical reactivity range of more than 6 orders of magnitude. When including the previously not described geometric isomer *Z*-α-*p*-OMe-C_6_H_4_-TMC the difference in the k_2_ values between the most electrophilic compound α-CN-TMC and *Z*-α-*p*-OMe-C_6_H_4_-TMC is even 300 million fold, since we determined the k_2_ value for *Z*-α-*p*-OMe-C_6_H_4_-TMC to be 0.0000196 ± 0.0000021 M^-1^s^-1^.

We examined whether the chalcones exert cytoprotective effects in a model of staurosporine-induced apoptosis in RAW264.7 macrophages. Initiation of apoptosis is inhibited by phosphorylation of apoptosis substrates such as caspases. Staurosporine is a broad-spectrum protein kinase inhibitor [26] and as such is utilized to induce the caspase-dependent mitochondrial apoptotic pathway. As a consequence annexin-V is bound on the outer site of the membrane. Exposure to staurosporine increased the percentage of annexin-V positive cells (mean over all groups = 27.9 ± 4.2%; p < 0.001). Pretreatment of RAW264.7 macrophages using α-X-TMCs with X = CN, Br, *p*-NO_2_-C_6_H_4_, I, H F, Me, Ph, NO_2_, CF_3_, COOEt, COOH; α-H-HC, calythropsin and 2’-hydroxychalcone had no diminishing influence on the staurosporine induced increase in annexin-V positive cells. α-Cl-TMC administration revealed a tendency to a reduced number of annexin-V positive cells, but failed to reach significance as was found for chalcone (see Figs 1 and 2).

*E*-α-*p*-OMe-C_6_H_4_-TMC pre-treatment demonstrated a significant reduction of annexin-V positive cells (Fig 1, 1 μM: 20.6 ± 3.2% and 10 μM: 17.1 ± 3.8%; p < 0.001). In contrast, its geometric isomer *Z*-α-*p*-OMe-C_6_H_4_-TMC showed only a small reduction of annexin-V positive cells after staurosporine-induced apoptosis at 10 μM, but this effect was not significant (Fig 2A, 22.9 ± 3.5%; p > 0.05).

### *E*-α-*p*-OMe-C_6_H_4_-TMC reduces staurosporine-induced apoptosis in a dose-dependent manner

**Fig 3:**
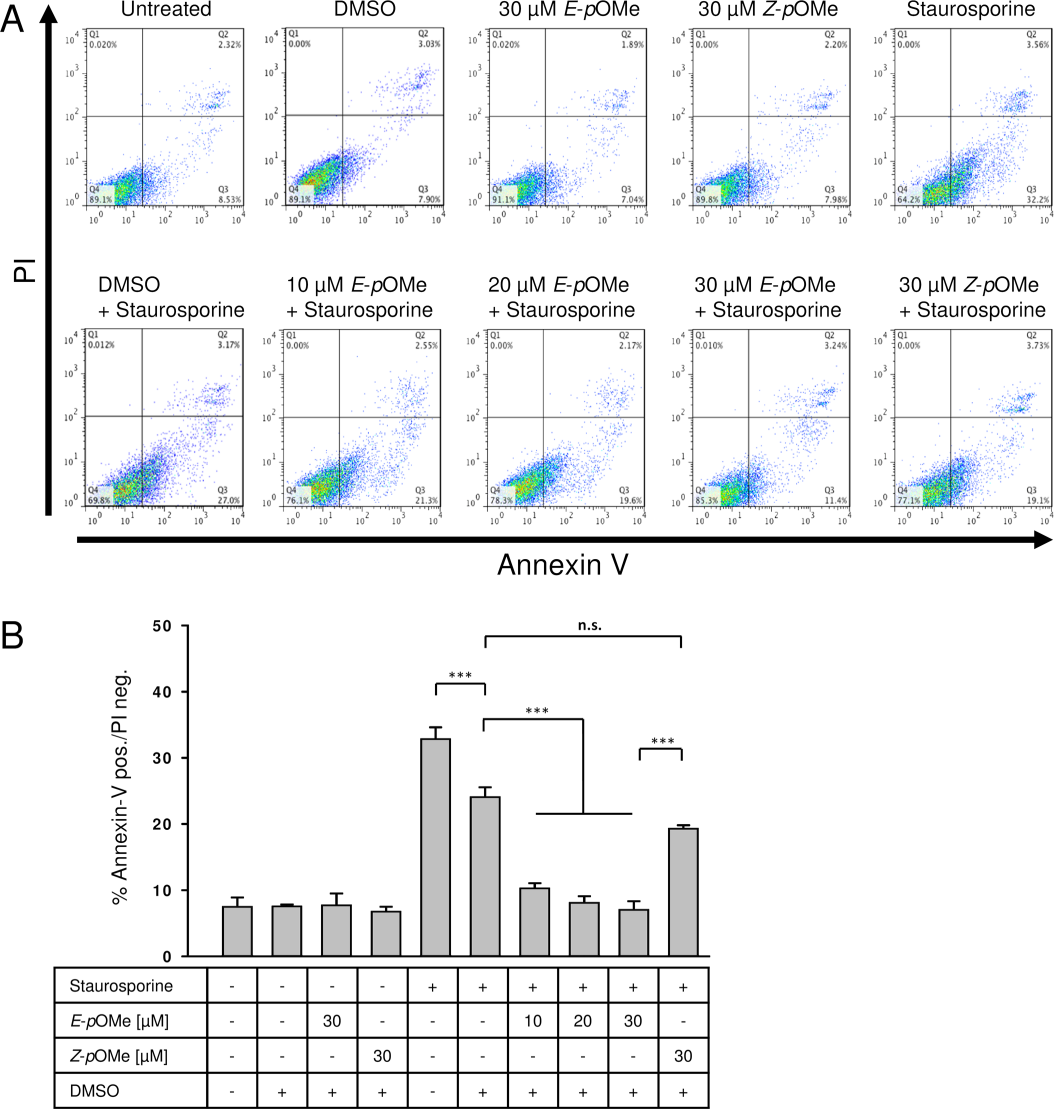
Flow cytometric analysis. RAW264.7 cells were pretreated with either *E*-α-*p*-OMe-C_6_H_4_-TMC or *Z*-α-*p*-OMe-C_6_H_4_-TMC (short: *E*-*p*OMe, *Z*-*p*OMe) for 3 h. Apoptosis was induced using staurosporine (1 μM for 2 h). A. Representative experiment after FITC annexin-V and propidium iodide (PI) staining. 1 × 10^4^ cells were analyzed in each experiment. B. Effect of *E*-α-*p*-OMe-C_6_H_4_-TMC and *Z*-α-*p*-OMe-C_6_H_4_-TMC on cytoprotection (n = 6; mean ± S.E.M.; *** = p < 0.001).

Compared to untreated cells, neither DMSO nor the chalcones *E*-α-*p*-OMe-C_6_H_4_-TMC or *Z*-α-*p*-OMe-C_6_H_4_-TMC (each 30 μM for 5 h) showed any increase in annexin-V positive cells compared to untreated cells (Fig 3, untreated 7.6 ± 1.3; DMSO 7.9 ± 0.04; 30 μM *E*-α-*p*-OMe-C_6_H_4_-TMC 7.8 ± 1.7; 30 μM *Z*-α-*p*-OMe-C_6_H_4_-TMC 6.8 ± 0.7% annexin-V positive / PI negative cells). Exposure of RAW264.7 cells to 1 μM staurosporine for 2 h increased the amount of annexin-V positive and propidium iodide (PI) negative cells (Fig 3 A and B, staurosporine 32.9 ± 1.7%) [27]. Pretreatment of RAW264.7 macrophages with *E*-α-*p*-OMe-C_6_H_4_-TMC shows a significant and dose dependent reduction in annexin-V positive / PI negative cells in the context of staurosporine-induced apoptosis (Fig 3A and 3B, staurosporine 32.9 ± 1.7 vs. staurosporine + 10 μM *E*-α-*p*-OMe-C_6_H_4_-TMC 10.4 ± 0.7; staurosporine + 20 μM *E*-α-*p*-OMe-C_6_H_4_-TMC 8.2 ± 0.9; staurosporine + 30 μμM *E*-α-*p*-OMe-C_6_H_4_-TMC 7.1 ± 1.2% annexin-V positive / PI negative cells, *** = p < 0.001). Although pretreatment with DMSO lead to a reduction of annexin-V positive / PI negative cells compared to staurosporine (Fig 3B, staurosporine 32.9 ± 1.7 vs. staurosporine + DMSO 21.7 ± 1.4% annexin-V positive / PI negative cells, *** = p < 0.001), this reduction differed significantly from the reduction achieved by the pretreatment with *E*-α-*p*-OMe-C_6_H_4_-TMC (Fig 3A and 3B, staurosporine + DMSO 21.7 ± 1.4 vs. staurosporine + 10/20/30 μM *E*-α-*p*-OMe-C_6_H_4_-TMC: 10.4 ± 0.7; 8.2 ± 0.9; 7.1 ± 1.2% annexin-V positive / PI negative cells, all *** = p < 0.001).

In contrast, pretreatment with 30 μM of the isomeric *Z*-α-*p*-OMe-C_6_H_4_-TMC prior to staurosporine treatment gave no significant reduction in annexin-V positive / PI negative cells compared to the pretreatment with DMSO + staurosporine (Fig 3A and 3B, staurosporine + DMSO 21.7 ± 1.4 vs. staurosporine + 30 μM *Z*-α-*p*-OMe-C_6_H_4_-TMC 19.7 ± 0.4% annexin-V positive / PI negative cells; not significant). The comparison of *E*- and *Z*-α-*p*-OMe-C_6_H_4_-TMC at equal amounts revealed their clear difference in activity (Fig 3A, and 3B, staurosporine + 30 μM *E*-α-*p*-OMe-C_6_H_4_-TMC 7.1 ± 1.2 vs. staurosporine + 30 μM *Z*-α-*p*-OMe-C_6_H_4_-TMC 19.7 ± 0.4% annexin-V positive / PI negative cells, *** = p < 0.001).

### *E*-α-*p*-OMe-C_6_H_4_-TMC exhibits no toxic effects

**Fig 4:**
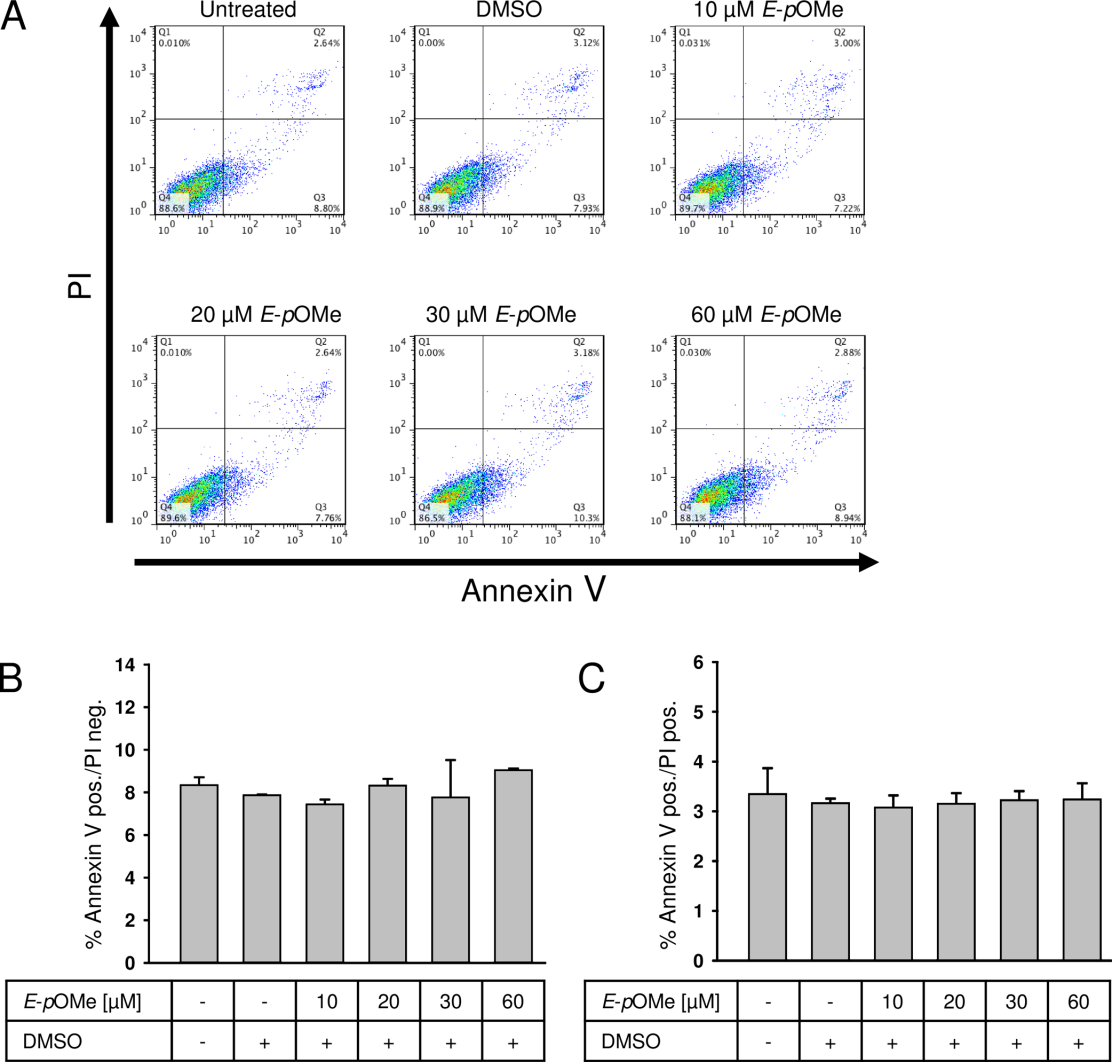
Flow cytometric analysis. RAW264.7 cells were treated with *E*-α-*p*-OMe-C_6_H_4_-TMC (short: *E*-*p*OMe) (10, 20, 30 and 60 μM) alone for 5 h. A. Representative experiment after FITC annexin-V and propidium iodide staining (PI). 1 × 10^4^ cells were analyzed in each experiment. B. Non-toxic effect of *E*-α-*p*-OMe-C_6_H_4_-TMC. Apoptotic cells (annexin-V positive/ PI negative) were analyzed (n = 4; mean ± S.E.M.). C. Non-toxic effect of *E*-α-*p*-OMe-C_6_H_4_-TMC. Necrotic cells (annexin-V positive / PI positive) were analyzed (n = 4; mean ± S.E.M.).

Exposure of RAW264.7 macrophages to the indicated concentrations of *E*-α-*p*-OMe-C_6_H_4_-TMC for 3 and 5 h did not demonstrate any toxic effects (Fig 4A–4C, displaying the results of 5 h pretreatment; 3 h data not shown).

To measure the toxicity of *E*-α-*p*-OMe-C_6_H_4_-TMC we incubated RAW264.7 macrophages for 5 h in medium containing a range of 10 to 60 μM *E*-α-*p*-OMe-C_6_H_4_-TMC. Analysis revealed no increase in apoptotic cells judged by annexin-V positive / PI negative characteristics (Fig 4B: untreated 8.4 ± 0.9 vs. 10 μM *E*-α-*p*-OMe-C_6_H_4_-TMC 7.5 ± 0.4 vs. 20 μM *E*-α-*p*-OMe-C_6_H_4_-TMC 8.3 ± 0.5 vs. 30 μM *E*-α-*p*-OMe-C_6_H_4_-TMC 7.8 ± 3.9 vs. 60 μM *E*-α-*p*-OMe-C_6_H_4_-TMC 9.1±0.1% annexin-V positive / PI negative cells, not significant). Also, no necrotic cells were found as assigned with annexin-V positive / PI positive staining (Fig 4C: untreated 3.4 ±0 .1 vs. 10 μM *E*-α-*p*-OMe-C_6_H_4_-TMC 3.1 ± 0.5 vs. 20 μM *E*-α-*p*-OMe-C_6_H_4_-TMC 3.2 ± 0.4 vs. 30 μM *E*-α-*p*-OMe-C_6_H_4_-TMC 3.2 ± 0.3 vs. 60 μM *E*-α-*p*-OMe-C_6_H_4_-TMC 3.2 ± 0.6% annexin-V positive / PI positive cells, not significant).

### *E*-α-*p*-OMe-C_6_H_4_-TMC induces HO-1 protein expression in a dose-dependent manner

**Fig 5:**
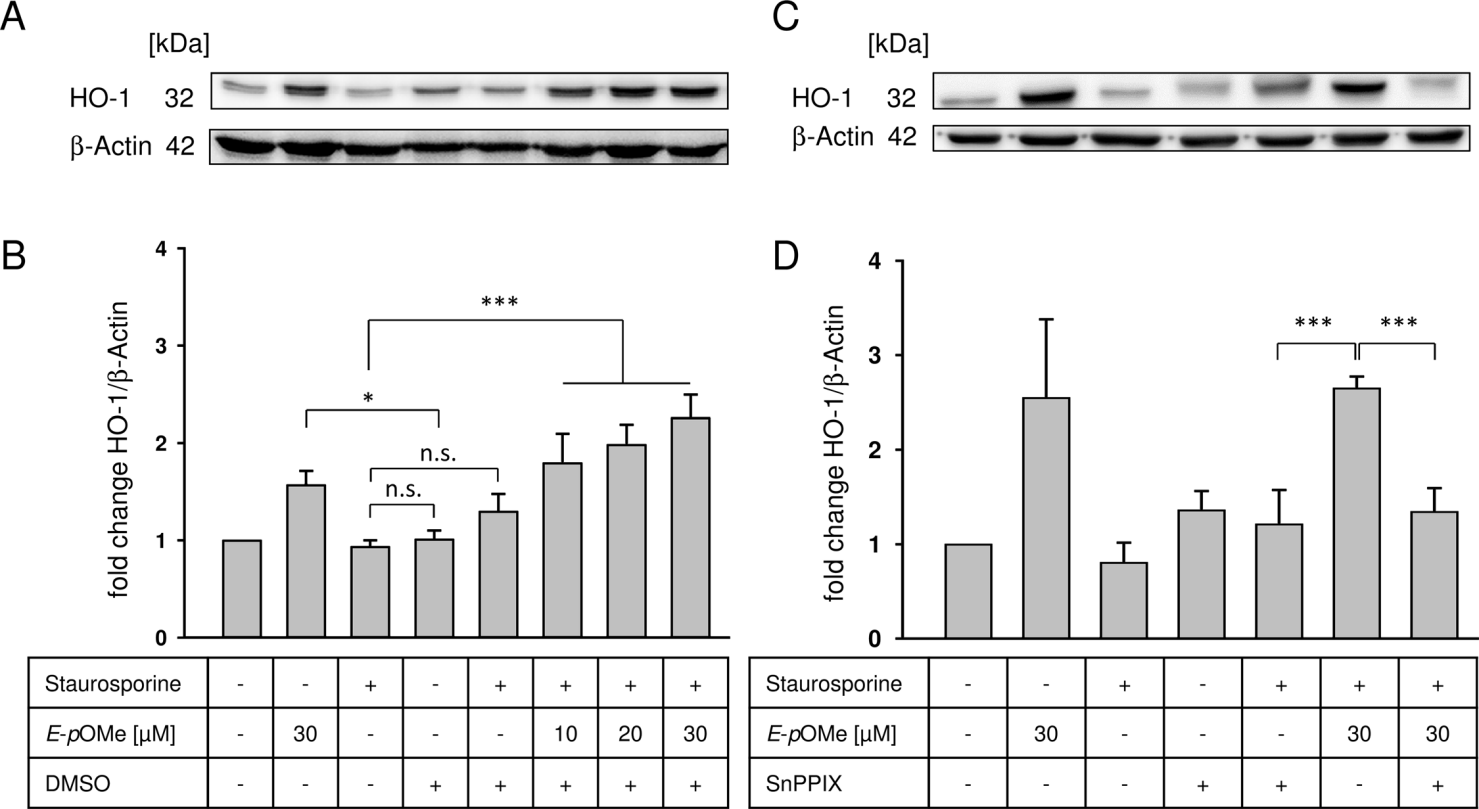
Western Blot analysis. A. RAW264.7 cells were pretreated with *E*-α-*p*-OMe-C_6_H_4_-TMC (short: *E*-*p*OMe) for 3 h, apoptosis was induced using staurosporine (1 μM for 2 h). Whole-cell extracts were prepared and HO-1 protein expression levels were analyzed. The image is representative of five independent experiments that showed similar results. B. Densitometric analysis of A, optical density of HO-1 normalized against β-actin (n = 5; mean ± S.E.M.; *** = p < 0.001; * = p < 0.05); C. RAW264.7 cells were pretreated with SnPP-IX (80 μM for 1 h) to inhibit HO-1 expression prior to the treatment with *E*-α-*p*-OMe-C_6_H_4_-TMC for 3 h, apoptosis was induced using staurosporine (1 μM for 2 h). Whole-cell extracts were prepared and HO-1 protein expression levels were analyzed. The image is representative of five independent experiments that showed similar results. D. Densitometric analysis of C, optical density of HO-1 normalized against β-actin (n = 5; mean ± S.E.M.; *** = p < 0.001).

RAW264.7 macrophages treated with 30 μM *E*-α-*p*-OMe-C_6_H_4_-TMC without induction of apoptosis showed a significant increase in HO-1 protein expression compared to cells treated with DMSO alone (Fig 5B: DMSO 1.01 ± 0.13 vs. 30 μM *E*-α-*p*-OMe-C_6_H_4_-TMC 1.76 ± 0.33 fold change HO-1/β-actin; n = 5; * = p < 0.05). Treating cells only with staurosporine or in combination with DMSO had no effect on HO-1 protein expression (Fig 5B; staurosporine 1.01 ± 0.19 vs. staurosporine + DMSO 1.3 ± 0.4 fold change HO-1/β-actin; not significant). Since *E*-α-*p*-OMe-C_6_H_4_-TMC is dissolved in DMSO, pretreatment of macrophages with DMSO before induction of apoptosis served as a negative control. Pretreatment of cells with indicated concentrations of *E*-α-*p*-OMe-C_6_H_4_-TMC for 3 h prior to induction of apoptosis resulted in a dose dependent and significant increase of HO-1 protein expression (Fig 5A, lanes 3 and 5 vs. 6, 7 and 8 and Fig 5B, staurosporine 1.01 ± 0.19 and staurosporine + DMSO 1.3 ± 0.4 vs. staurosporine + 10/20/30 M *E*-α-*p*-OMe-C_6_H_4_-TMC: 1.8 ± 0.6; 2.2 ± 0.54 and 2.26 ± 0.8 fold change HO-1/β-actin, * = p < 0.05; *** = p < 0.001).

To proof if HO-1 protein expression is *E*-α-*p*-OMe-C_6_H_4_-TMC-dependent, we analyzed whether blocking HO-1 using the HO-1 inhibitor SnPP-IX abolishes the effect of *E*-α-*p*-OMe-C_6_H_4_-TMC. Cells pretreated with SnPP-IX prior to 30 μM *E*-α-*p*-OMe-C_6_H_4_-TMC and staurosporine treatment showed a significant reduction of the HO-1 protein expression (Fig 5C, lanes 6 vs. 7, Fig 5D, staurosporine + 30 μM *E*-α-*p*-OMe-C_6_H_4_-TMC 2.65 ± 0.12 vs. SnPP-IX + staurosporine + 30 μM *E*-α-*p*-OMe-C_6_H_4_-TMC 1.34 ± 0.25 fold change HO-1/β-actin; *** = p < 0.001).

### *E*-α-*p*-OMe-C_6_H_4_-TMC induces HO-1 activity and exerts a HO-1 dependent cytoprotection

**Fig 6:**
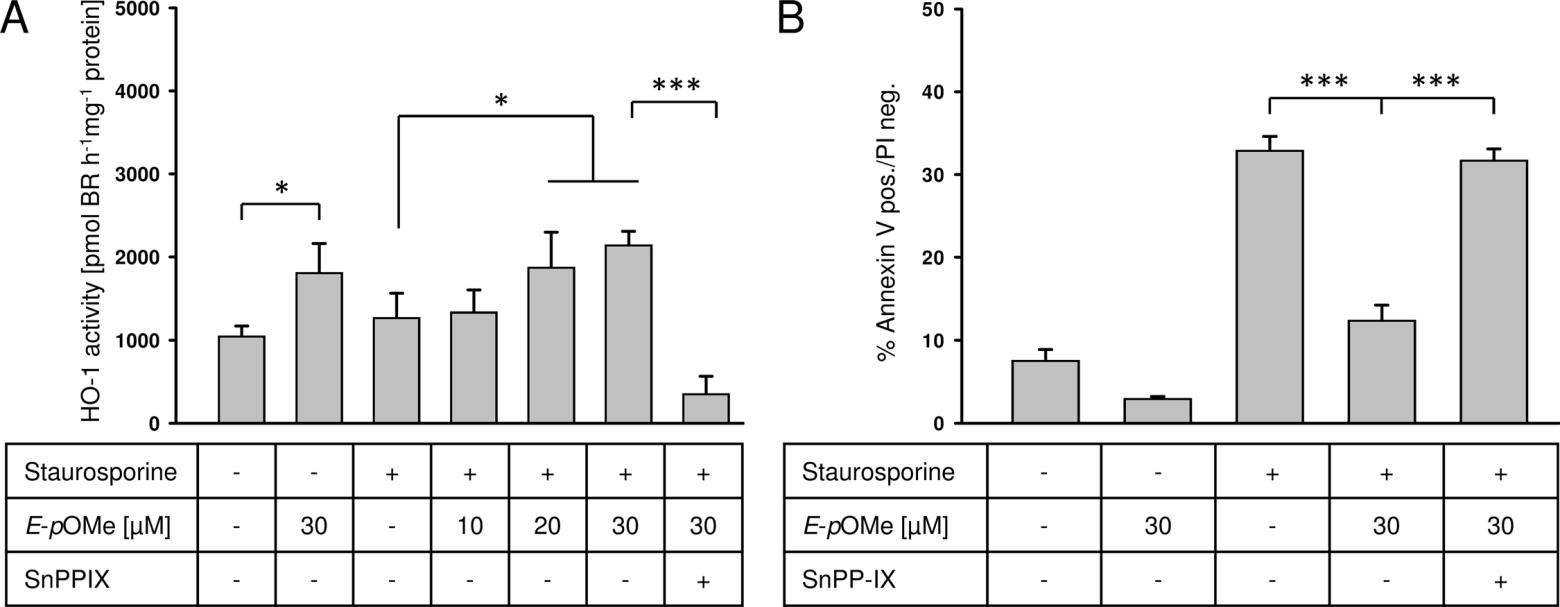
A. HO-1 activity assay. RAW264.7 cells were pretreated with SnPP-IX (80 μM for 1 h) to inhibit HO-1 activity prior to the treatment *E*-α-*p*-OMe-C_6_H_4_-TMC (short: *E*-*p*OMe) for 3 h, apoptosis was induced using staurosporine (1 μM for 2 h). Whole-cell protein extracts were prepared and HO activity was measured by ELISA-based bilirubin quantification; B. Flow cytometric analysis. RAW264.7 cells were pretreated with SnPP-IX (80 μM for 1 h) to inhibit HO-1 activity prior to the treatment with *E*-α-*p*-OMe-C_6_H_4_-TMC for 3 h, apoptosis was induced using staurosporine (1 μM for 2 h). Cells were stained with FITC annexin-V and propidium iodide to mark apoptotic cells. 1 × 10^4^ cells were analyzed in each experiment (n = 4; mean ± S.E.M.; * = p < 0.05).

Apart from the HO-1 protein expression, HO-1 activity was analyzed. Cells treated with 30 μM *E*-α-*p*-OMe-C_6_H_4_-TMC without induction of apoptosis demonstrated a significant increase in HO-1 activity compared to untreated cells (Fig 6A, untreated 1050 ± 339 vs. 30 μM *E*-α-*p*-OMe-C_6_H_4_-TMC 1780 ± 153 HO activity [pmol BR h^-1^mg^-1^ protein]; * = p < 0.05).

Similarly to the results obtained for HO-1 protein expression, pretreatment of cells with increasing concentrations of *E*-α-*p*-OMe-C_6_H_4_-TMC for 3 h and induction of apoptosis resulted in a dose dependent and significant increase of HO-1 activity (Fig 6A, staurosporine 1270 ± 583 vs. staurosporine + 10, 20, 30 μM *E*-α-*p*-OMe-C_6_H_4_-TMC 1340 ± 530, 1870 ± 845 and 2140 ± 331 HO-1 activity [pmol BR h^-1^mg^-1^ protein]; * = p < 0.05). Using SnPP-IX, prior to *E*-α-*p*-OMe-C_6_H_4_-TMC and staurosporine treatment abolished the activity of HO-1 (Fig 6A, staurosporine + 30 μM *E*-α-*p*-OMe-C_6_H_4_-TMC 2140 ± 331 vs. SnPP-IX + staurosporine + 30 μM *E*-α-*p*-OMe-C_6_H_4_-TMC 144 ± 35 HO-1 activity [pmol BR h^-1^mg^-1^ protein]; *** = p < 0.001).

To answer the question, if *E*-α-*p*-OMe-C_6_H_4_-TMC-dependent HO-1 induction is responsible for cytoprotection, we analyzed the percentage of annexin-V positive / PI negative cells with and without SnPP-IX (Fig 6B). While staurosporine-induced apoptosis was significantly reduced by pretreating cells with *E*-α-*p*-OMe-C_6_H_4_-TMC, this effect was abolished using SnPP-IX (Fig 6B, staurosporine + 30 μM *E*-α-*p*-OMe-C_6_H_4_-TMC 13.1 ± 1.4 vs. SnPP-IX + staurosporine + 30 μM *E*-α-*p*-OMe-C_6_H_4_-TMC 31.7 ± 2.7% annexin-V positive / PI negative cells; *** = p < 0.001)

### *E*-α-*p*-OMe-C_6_H_4_-TMC reduces the formation of reactive oxygen species

**Fig 7:**
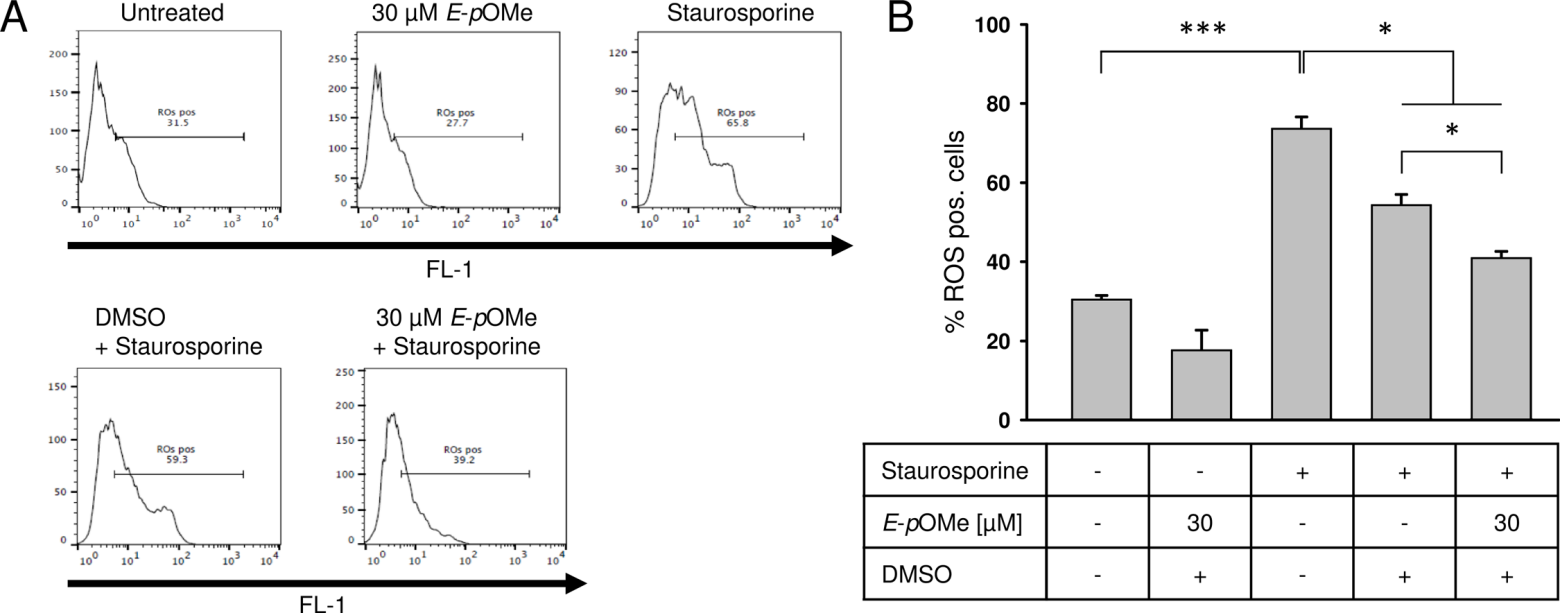
Total ROS detection by flow cytometric analysis. RAW264.7 cells were pretreated with *E*-α-*p*-OMe-C_6_H_4_-TMC (short: *E*-*p*OMe) for 3 h, ROS production was induced using staurosporine (1 μM for 2 h); A. Representative experiment after intracellular ROS staining. 1 × 10^4^ cells were analyzed in each experiment; B. *E*-α-*p*-OMe-C_6_H_4_-TMC mediated effect on intracellular ROS production (n = 6; mean ± S.E.M.; * = p < 0.05).

ROS production was induced using staurosporine. Compared to untreated cells application of staurosporine lead to a significant increase in ROS production (Fig 7A, and 7B, untreated 30.5 ± 0.9 vs. staurosporine 73.7 ± 6.9% ROS positive cells; *** = p < 0.001) Pretreatment with *E*-α-*p*-OMe-C_6_H_4_-TMC reduced ROS formation significantly (Fig 7A and 7B, staurosporine 73.7 ± 6.9 vs. staurosporine + 30 μM *E*-α-*p*-OMe-C_6_H_4_-TMC 40.9 ± 1.5% ROS positive cells; * = p < 0.05). To exclude the possibility that this effect is caused by DMSO we compared ROS production induced by staurosporine in cells pretreated with DMSO alone to cells pretreated with *E*-α-*p*-OMe-C_6_H_4_-TMC dissolved in DMSO (Fig 7A and 7B, staurosporine + DMSO 54.4 ± 2.5 vs. staurosporine + 30 μM *E*-α-*p*-OMe-C_6_H_4_-TMC 40.9 ± 1.5% ROS positive cells; * = p < 0.05).

### *E*-α-*p*-OMe-C_6_H_4_-TMC induces the translocation of Nrf2 and reduces NF-κB activity

**Fig 8:**
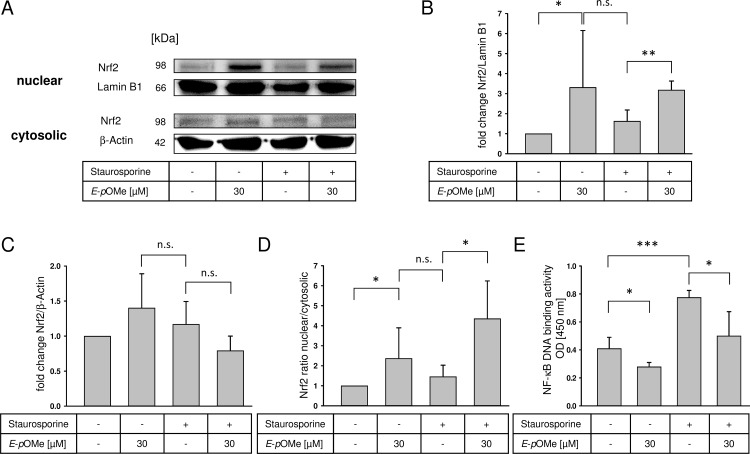
Effects of *E*-α-*p*-OMe-C_6_H_4_-TMC (short: *E*-*p*OMe) on Nrf2 expression after staurosporine treatment. RAW264.7 cells were pretreated with *E*-α-*p*-OMe-C_6_H_4_-TMC (30 μM) for 3 h, followed by staurosporine (1 μM for 2 h). Nuclear and cytosolic cell extracts were prepared and Nrf2 expression was measured by Western blot analysis. A. The image is representative of four independent experiments that showed similar results; B. Densitometric analysis of nuclear extracts, optical density of Nrf2 normalized against Lamin B1 (n = 4; mean ± S.E.M.; ** = p < 0.005); C. Densitometric analysis of cytosolic extracts, optical density of Nrf2 normalized against β-actin (n = 4; mean ± S.E.M.); D. Densitometric analysis of nuclear Nrf2 normalized to cytosolic Nrf2 (n = 4; mean ± S.E.M.; * = p < 0.05); E. Effects of *E*-α-*p*-OMe-C_6_H_4_-TMC on NF-κB DNA binding activity after staurosporine treatment. RAW264.7 cells were pretreated with *E*-α-*p*-OMe-C_6_H_4_-TMC (30 μM) for 3 h, followed by staurosporine (1 μM for 2 h). Nuclear extracts were prepared and NF-κB DNA binding activity was measured (n = 4; mean ± S.E.M.; * = p < 0.05).

To elucidate a possible mechanism leading to the proposed cytoprotective effects via HO-1 induction by *E*-α-*p*-OMe-C_6_H_4_-TMC, we analyzed the translocation of the transcription factor Nrf2. Untreated cells compared to cells treated with *E*-α-*p*-OMe-C_6_H_4_-TMC alone showed a significant translocation of Nrf2 into the nucleus (Fig 8A, Lane 1 vs. 2; Fig 8B, untreated normalized to 1 vs. 30 μM *E*-α-*p*-OMe-C_6_H_4_-TMC 3.3 ± 2.8 fold change Nrf2/Lamin B1, * = p < 0.05). We did not detect an increased Nrf2 translocation after staurosporine induction alone, whereas pretreatment with *E*-α-*p*-OMe-C_6_H_4_-TMC before induction of apoptosis caused a significant nuclear translocation of Nrf2 (Fig 8A and 8B, staurosporine 1.6 ± 0.5 vs. staurosporine + 30 μM *E*-α-*p*-OMe-C_6_H_4_-TMC 3.2 ± 0.4 fold change Nrf2/Lamin B1; ** = p < 0.01). The cytosolic extracts did not show any significant differences regarding Nrf2 expression levels (Fig 8A and 8C). Fig 8D demonstrates the nuclear to cytosolic ratio of Nrf2 and in this way combines the results highlighted in Fig 8B and 8C. Macrophages treated with *E*-α-*p*-OMe-C_6_H_4_-TMC revealed a significantly increased nuclear to cytosolic ratio of Nrf2 compared to untreated cells, whereas *E*-α-*p*-OMe-C_6_H_4_-TMC pretreatment before induction of apoptosis lead to a significant increased ratio compared to staurosporine alone (Fig 8D, untreated normalized to 1 vs. 30 μM *E*-α-*p*-OMe-C_6_H_4_-TMC 2.4 ± 1.5; staurosporine 1.5 ± 0.6 vs. staurosporine + 30 μM *E*-α-*p*-OMe-C_6_H_4_-TMC 4.3 ± 1.9 Nrf2 ratio nuclear/cytosolic, * = p < 0.05).

To analyze NF-κB DNA binding activity, we prepared nuclear extracts after pretreating macrophages with *E*-α-*p*-OMe-C_6_H_4_-TMC and staurosporine. In contrast to the results of Nrf2 expression, treatment with *E*-α-*p*-OMe-C_6_H_4_-TMC alone lead to a significantly decreased binding activity of NF-κB compared to untreated cells (Fig 8E, untreated 0.41 ± 0.08 vs. 30 μM *E*-α-*p*-OMe-C_6_H_4_-TMC 0.28 ± 0.03 NF-κB DNA binding activity OD [450 nm]; * = p < 0.05). Staurosporine caused a significantly increased binding activity of NF-κB compared to untreated cells. This effect is significantly reduced when cells are pretreated with *E*-α-*p*-OMe-C_6_H_4_-TMC prior to the induction of apoptosis (Fig 8E, staurosporine 0.77 ± 0.05 vs. 30 μM *E*-α-*p*-OMe-C_6_H_4_-TMC + staurosporine 0.57 ± 0.17 NF-κB DNA binding activity OD [450 nm]; * = p < 0.05).

### The two double bond isomers *E*-α-*p*-OMe-C_6_H_4_-TMC and *Z*-α-*p*-OMe-C_6_H_4_-TMC possess significantly different 3-dimentional structures

**Fig 9:**
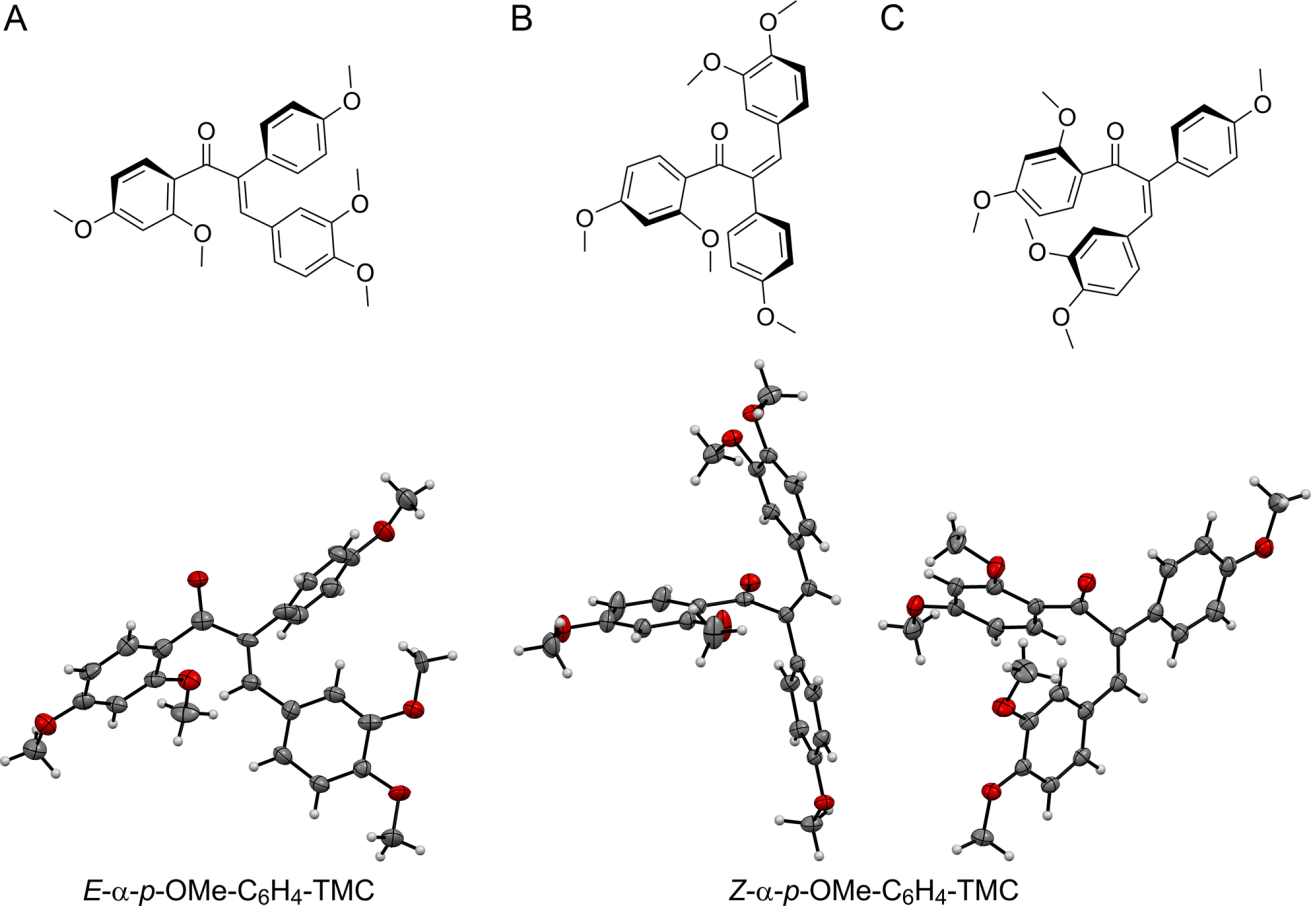
Solid-state structures from X-ray analysis of: A. *E*-α-*p*-OMe-C_6_H_4_-TMC. One out of four, essentially the same, molecules from the unit cell is shown. B,C. *Z*-α-*p*-OMe-C_6_H_4_-TMC. The two different conformers from the unit cell are shown. Ellipsoids are drawn at the 50% probability levels.

From the X-ray analysis of the two solid state structures it becomes clear that both geometric isomers adopt two very different structures. While *E*-α-*p*-OMe-C_6_H_4_-TMC (Fig 9A) is found in the *s*-trans conformation (Fig 9A), its isomer *Z*-α-*p*-OMe-C_6_H_4_-TMC crystalizes in an intermediate form which is in between the *s*-cis and *s*-trans conformation (Fig 9B and 9C). For *Z*-α-*p*-OMe-C_6_H_4_-TMC it appears that the B-ring of the chalcone scaffold and the *p*-methoxyphenyl X substituent are in one plane with the 2,3 double bond; and, in that sense form a trans-stilbene-like unit. Moreover, the plane of the chalcone A-ring together with the carbonyl carbon is placed orthogonal to the trans-stilbene-like unit forming the main conjugated system.

*E*-α-*p*-OMe-C_6_H_4_-TMC on the other hand has a very similar solid state structure compared to the α-X-TMC halogen compounds with X = F, Cl, Br and I [21], which also are found in the *s*-trans conformation and proved to be very good to moderate electrophiles.

## Discussion

To date, many natural products with α,β-unsaturated carbonyl units (i.e. natural chalcones and flavonoids) are described as antioxidants with beneficial biological potential [28, 29]. We tested some α-H-chalcones, some with free phenolic hydroxyl groups, together with a previously synthesized library of α-X-substituted tetramethoxychalcones (α-X-TMCs) to examine their therapeutic potential. For the α-X-TMCs it was particularly important to survey their activity depending on the substituents in the α-position which directly influence their electrophilicity, but also 3D-structure. Among the 14 α-X-TMCs, *E*-α-*p*-OMe-C_6_H_4_-TMC displayed the most promising potential as a new therapeutic agent, demonstrating a highly significant reduction of annexin-V positive cells. All other tested chalcones showed either no protection or even an increase in apoptosis.

Pretreatment of RAW264.7 macrophages with 10, 20 or 30 μM of *E*-α-*p*-OMe-C_6_H_4_-TMC induced a significant dose-dependent reduction of apoptotic cells after staurosporine treatment. Applying higher concentrations of *E*-α-*p*-OMe-C_6_H_4_-TMC up 1000 μM did not lead to any additional anti-apoptotic effect. To challenge the non-cytotoxic properties of *E*-α-*p*-OMe-C_6_H_4_-TMC, we doubled the concentration with the highest anti-apoptotic effect without an increase of apoptosis or necrosis. As pretreatment with *Z*-α-*p*-OMe-C_6_H_4_-TMC did not show a significant antiapoptotic effect compared to the pretreatment with DMSO, we cannot rule out that the observed antiapoptotic trend of *Z*-α-*p*-OMe-C_6_H_4_-TMC is caused by the solvent DMSO. Alternatively, it might be possible that small amounts of the other double bond isomer are being formed during handling of solutions or in the cell to contribute to this minor effect.

After verification of its anti-apoptotic effect we examined the upregulation and activity of cytoprotective enzymes whereby HO-1 overexpression and activity showed a highly significant dose-dependency. As a proof of principle, inhibition of HO-1 abolished the antiapoptotic effect of *E*-α-*p*-OMe-C_6_H_4_-TMC. In recent years many *in-vitro* and *in-vivo* studies have been conducted on CO application as a gas or intravenous [30–32]. For intravenous application CO may be linked to metal complexes (CO-RMs) [13, 33, 34]. In general HO-1 cytoprotection is not exclusively dependent on CO and on biliverdin/bilirubin. Moreover, HO-1 acts as an antioxidant reducing the cellular pool of free heme and iron [35]. We are convinced that non-cytotoxic HO-1 induction is more potent in cyto- and organ protection then just imitating its natural activity by adding biliverdin, bilirubin or CO.

Therapeutic strategies aimed at utilizing the cytoprotective effects of HO-1 are hindered by the fact that most pharmacological inducers negatively influence organ function by themselves and are not available for application in patients because of their immense toxicity and undesirable side effects [36]. As a consequence *in-vitro* and *in-vivo* studies utilizing drugs that are applied in daily clinical practice were conducted. Inhalation anesthetics like sevoflurane and isoflurane were used to induce HO-1 *in-vitro* and *in-vivo*; a potential problem concerning these studies is the fact that the high concentrations used in the experiments are far away from the concentrations applied in daily clinical practice [37–39]. It is most tempting to speculate about *in-vivo* effects of *E*-α-*p*-OMe-C_6_H_4_-TMC. Since *in-vitro* concentrations of 10 to 1000 μM have shown no toxic or any other adverse effects, the possible therapeutic range is huge. As no concentration have been tested in animals so far, dose dependency of *E*-α-*p*-OMe-C_6_H_4_-TMC concerning HO-1 induction must be tested individually.

Antioxidant activity refers to the prevention or retardation of the oxidation of other molecules, which is usually caused by reactive oxygen species. *E*-α-*p*-OMe-C_6_H_4_-TMC decreased ROS levels after induction of apoptosis. To exclude the possibility that the reduction of ROS is caused by the solvent DMSO, pretreatment with DMSO before staurosporine induction was used as negative control. Since *E*-α-*p*-OMe-C_6_H_4_-TMC is a relatively electron-rich compound it could be argued that it might act as a direct reducing agent to neutralize radicals. Due to the lack of free hydroxyl groups, which are typical for natural antioxidative flavonoids such as chalcones, that is less likely, but a radical entrapment through the enone system can be envisioned.

Finally, we examined whether HO-1 induction is caused by an increased Nrf2 activation. Nrf2 plays a crucial role in the expression of cytoprotective enzymes among which HO-1 is one of the most important ones [18]. In its inactivated form Nrf2 is bound to its inhibitor Keap1. Certain stimuli lead to a release of Nrf2 from its inhibitor Keap1 resulting in translocation of Nrf2 into the nucleus [40, 41]. Our results show a significant nuclear translocation of Nrf2 after *E*-α-*p*-OMe-C_6_H_4_-TMC treatment. Further studies are needed to examine the exact mechanism that leads to the described translocation.

The transcription factor NF-κB is another central key player looking at apoptosis and its mechanisms [42, 43]. It is essential for the function of our immune system and is responsible for the expression of cytokines, growth factors and proapoptotic factors. According to present studies, the function of NF-κB regarding apoptosis remains inconsistent. There are many atypical inducers of apoptosis which act through NF-κB activation, such as UV radiation, H_2_O_2_ or some anticancer drugs [44]. Apart from that inhibition of NF-κB activation leads to neuroprotection [45]. In addition, HO-1 seems to be a down-modulator of NF-κB activation without affecting the expression of cytoprotective genes [46]. Our results show that *E*-α-*p*-OMe-C_6_H_4_-TMC treatment and pretreatment just prior to induction of apoptosis leads to a significant reduction of NF-κB activity.

In our previous work using 1 or 5 μM *E*-α-*p*-OMe-C_6_H_4_-TMC we were not able to prove any anti-inflammatory effects in RAW264.7 cells based on Nrf2-activation [22]. Using now higher concentrations for both *E*- and its newly synthesized isomer *Z*-α-*p*-OMe-C_6_H_4_-TMC, only the *E*-isomer proved antiapoptotic and anti-inflammatory effects via Nrf2-, NF-κB- and ROS-dependent pathways. Since we could demonstrate that both isomers can act as electrophiles, but are very different in their electrophilicity—namely by a factor of 440 less for the *Z*-isomer—it could be argued that the *Z*-isomer is a too weak electrophile to make use of this chemical property within the cell. That could be one reason for the lack of activity in *Z*-α-*p*-OMe-C_6_H_4_-TMC. On the contrary for *E*-α-*p*-OMe-C_6_H_4_-TMC it can be argued that its biological activity might indeed depend in part on the intrinsic alkylation power of the Michael acceptor unit present in its enone system. But, since its k_2_ value is very low the often feared electrophilicity-related non-specific toxicity does not come into play and opens the door for a potential clinical use.

Moreover, since one has to assume that the third aromatic ring is essential to provide more interactions, it is worthwhile to look closer at the other two 1,2,3-triaryl-compounds in our library, namely α-*p*-NO_2_-C_6_H_4_-TMC and α-Ph-TMC (both are *E*-isomers). Despite their pronounced difference in their electrophilicities both are neither toxic nor do they reduce staurosporine-induced apoptosis. This indicates that the methoxy group in *E*-α-*p*-OMe-C_6_H_4_-TMC plays an important role creating non-covalent binding interactions.

To understand the quite pronounced difference in the chemical reactivity of *E*-α-*p*-OMe-C_6_H_4_-TMC vs. *Z*-α-*p*-OMe-C_6_H_4_-TMC one can draw some analogies from the X-ray structures of the α-halogen-TMCs. Their electrophile behavior mainly depends on the electronic nature of the halogen substituents. More precisely, upon a weakening of the resonance effect due to the longer halogen bonds from F, Cl to Br the inductive effect lead to a better electrophilicity in Br than in Cl and in α-F-TMC. The more sterically demanding iodo substituent gave a more twisted conjugated system which lowered the kinetic activity. This effect can be anticipated in a similar way for *Z*-α-*p*-OMe-C_6_H_4_-TMC, but with a much stronger disruption of the conjugation of the enone system towards a main conjugation via the trans-stilbene unit involving the X-aromatic group and the B-ring. This reduced conjugation with the carbonyl carbon caused a very weak electrophilicity for *Z*-α-*p*-OMe-C_6_H_4_-TMC. Furthermore, the great differences in the 3D-structure most likely play a distinct role when binding to a particular site is required for activity. The alkylation ability might kick in once a certain residence time is reached at a potential alkylation site. In *E*-α-*p*-OMe-C_6_H_4_-TMC this potential interplay seems to be balanced in a way so that unspecific toxicity does not become relevant.

In conclusion, we utilized 19 chalcones out of which one shows a highly significant antiapoptotic effect in RAW264.7 macrophages in a dose-dependent manner. We demonstrate that this effect is caused by a dose-dependent induction of HO-1 via Nrf2 presenting one of the key mechanisms in cyto- and organ protection. Based on our results we are convinced that *E*-α-*p*-OMe-C_6_H_4_-TMC provides promising therapeutic potential for cyto- or even organ protection. Further studies are needed to verify our results in *in-vivo* models addressing these questions.

## Supporting Information

S1 Supporting Data Experimental details, characterization, and NMR spectra of *Z*-α-*p*-OMe-C_6_H_4_-TMC together with X-ray structures of *E*-α-*p*-OMe-C_6_H_4_-TMC and of *Z*-α-*p*-OMe-C_6_H_4_-TMC. (DOCX)

## Abbreviations

*E*-α-*p*-OMe-C_6_H_4_-TMC: *E*-α-(4-methoxyphenyl)-2’,3,4,4'-tetramethoxychalcone
HO-1: heme oxygenase-1
ROS: reactive oxygen species
SnPP-IX: tin protoporphyrin-IX
Nrf2: nuclear related factor 2
NF-κB: nuclear factor-κB
CO-RM: CO releasing molecule
DMSO: dimethylsulfoxid
PBS: phosphate buffered saline
BR: bilirubin
SDS: sodium dodecyl sulfate
BVR: biliverdin reductase
NADPH: nicotinamide adenine dinucleotide phosphate (reduced form)
ARE/EpRE: antioxidant/electrophile response element
