# Supplementary material for: The Cytoprotective Effects of E-α-(4-Methoxyphenyl)-2’,3,4,4'-Tetramethoxychalcone (E-α-p-OMe-C6H4-TMC)—A Novel and Non-Cytotoxic HO-1 Inducer
Source: PLoS One. 2015 Nov 13;10(11):e0142932. doi: 10.1371/journal.pone.0142932 (PMC4643879; doi:10.1371/journal.pone.0142932)
Supplement: S1 Supporting Data — (DOCX) [file pone.0142932.s001.docx]

**Supporting Information**

**The cytoprotective effects of *E*-α-(4-methoxyphenyl)-2’,3,4,4'-tetra­methoxychalcone (*E*-α-*p*-OMe-C_6_H_4_-TMC) - A novel and non-cytotoxic HO-1 inducer**

Kai B. Kaufmann,^1^ Nafisah Al-Rifai,^2^ Felix Ulbrich,^1^ Nils Schallner,^1^ Hannelore Rücker,^2^ Monika Enzinger,^2^ Hermina Petkes,^2^ Sebastian Pitzl,^2,3^ Ulrich Goebel,^1,*^ Sabine Amslinger^2,*^

^1^Department of Anesthesiology and Intensive Care Medicine, Freiburg, University Medical Center Freiburg, Germany; ^2^Institute of Organic Chemistry, University of Regensburg, Regensburg, Germany; and ^3^Institute of Pharmaceutical Biology, University of Regensburg, Regensburg, Germany

**Table of Contents**

[General experimental information 2](#_Toc432689295)

[Analytical data of *Z*-α-(4-methoxyphenyl)-2’,3,4,4'-tetramethoxychalcone 2](#_Toc432689296)

[X-Ray data of (*E*)-α-(4-methoxyphenyl)-2’,3,4,4'-tetramethoxychalcone and (*Z*)-α-(4-methoxyphenyl)-2’,3,4,4'-tetramethoxychalcone 3](#_Toc432689297)

[Supporting Table S1: Parameters of kinetic measurements 5](#_Toc432689298)

[^1^H NMR spectrum of *Z*-α-*p*-OMe-C_6_H_4_-TMC in CDCl_3_ at 400 MHz 6](#_Toc432689299)

[^13^C NMR spectrum of *Z*-α-*p*-OMe-C_6_H_4_-TMC in CDCl_3_ at 101 MHz 6](#_Toc432689300)

General experimental information

All reactions were carried out under N_2_ atmosphere in oven-heated glassware (110 °C) when dry conditions were required, and monitored by TLC on silica gel plates 60 F_254_ by Merck (Germany). Spots were detected under UV light (λ = 254 and 366 nm) or visualized by staining with vanillin/H_2_SO_4_ (6.0 g vanillin in 100 mL 95% EtOH/conc. H_2_SO_4_ 100:1). Column chromatography was performed on silica gel Geduran Si 60 (0.063-0.200 mm) by Merck. Preparative plates were prepared using silica gel 60 GF_254_ by Merck. Melting points are determined with an automated melting point system (OptiMelt apparatus, USA) and were uncorrected. IR spectroscopy was carried on a Specac Golden Gate Diamond Single Reflection ATR System Excalibur Series FTS3000MX by Bio-Rad (Germany). NMR spectra were recorded on Bruker spectrometer (USA): Avance 300. ^1^H NMR spectra are referenced to CDCl_3_ (7.26 ppm); ^13^C NMR spectra to CDCl_3_ (77.0 ppm). The following abbreviations are used to explain the multiplicities: s, singlet; d, doublet; dd, doublet of doublets; m, multiplet. Mass spectra were obtained on Agilent Technologies 6540 UHD (USA). The samples for X-ray analysis were recrystallized from EtOAc/hexanes and DCM/hexanes by vapor diffusion technique. All reagents were purchased from commercial sources and were used without further purification. Solvents were distilled before use and dried if water-free conditions were necessary

Analytical data of *Z*-α-(4-methoxyphenyl)-2’,3,4,4'-tetramethoxychalcone

***Z*-α-(4-Methoxyphenyl)-2’,3,4,4'-tetramethoxychalcone (*Z*-α-*p*-OMe-C_6_H_4_-TMC)**

Yellow solid, mp 102-103 ºC; ^1^H NMR (400 MHz, CDCl_3_): δ = 7.91 (d, *J* = 8.8 Hz, 1H), 7.36 (m, 2H), 6.85 (m, 5H), 6.70 (d, 1H, *J* = 8.8 Hz), 6.42 (dd, 1H, *J* = 8.8, 2.3 Hz), 6.35 (d, 1H, *J* = 2.3 Hz), 3.81 (s, 3H), 3.79 (s, 3H), 3.78 (s, 3H), 3.74 (s, 3H), 3.68 (s, 3H) ppm; ^13^C NMR (101 MHz, CDCl_3_): δ = 196.5, 165.1, 161.8, 159.2, 148.4, 148.3, 142.3, 134.6, 131.1, 129.4, 127.6 (2C), 126.1, 121.8, 120.3, 113.9 (2C), 111.4, 110.8, 105.2, 98.6, 55.7, 55.6, 55.4 (2C), 55.2 ppm; IR (neat): 3001, 2937, 2839, 1640, 1592, 1510, 1481, 1245, 1208, 1178, 1021, 958, 891, 828, 768 cm^-1^; MS (ESI) *m*/*z* (%): 435.18 [MH^+^] (100), 257.58 (1); HRMS (ESI): calcd. for C_26_H_26_O_6_ [MH^+^] 435.1808; found 435.1802.

X-Ray data of (*E*)-α-(4-methoxyphenyl)-2’,3,4,4'-tetramethoxychalcone and (*Z*)-α-(4-methoxyphenyl)-2’,3,4,4'-tetramethoxychalcone

**(*E*)-α-(4-methoxyphenyl)-2’,3,4,4'-tetramethoxychalcone (*E*-α-*p*-OMe-C_6_H_4_-TMC)**

CCDC no.: 1413001

The dihedral angle between the two aromatic rings (A-ring and B-Ring) is: 69.61°.

*Crystal data and structure refinement*

| Empirical formula | C_26_H_26_O_6_ |
| --- | --- |
| Formula weight | 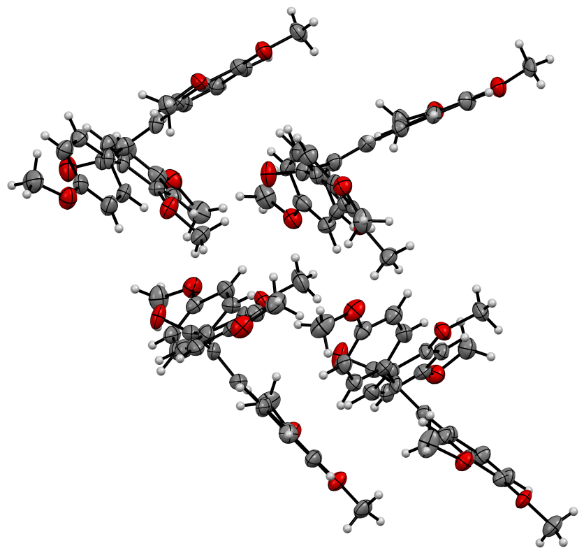434.47 |
| Temperature/K | 123.02(10) |
| Crystal system | monoclinic |
| Space group | Pn |
| a/Å | 13.1353(5) |
| b/Å | 13.0590(4) |
| c/Å | 25.9867(9) |
| α/° | 90 |
| β/° | 94.908(4) |
| γ/° | 90 |
| Volume/Å^3^ | 4441.3(3) |
| Z | 8 |
| ρ_calc_g/cm^3^ | 1.300 |
| μ/mm^‑1^ | 0.753 |
| F(000) | 1840.0 |
| Crystal size/mm^3^ | 0.16 × 0.14 × 0.05 |
| Radiation | CuKα (λ = 1.54184) |
| 2Θ range for data collection/° | 7.304 to 127.98 |
| Index ranges | -15 ≤ h ≤ 14, -15 ≤ k ≤ 15, -29 ≤ l ≤ 29 |
| Reflections collected | 8480 |
| Independent reflections | 8480 [R_int_ = N/A, R_sigma_ = 0.0228] |
| Data/restraints/parameters | 8480/2/1174 |
| Goodness-of-fit on F^2^ | 1.044 |
| Final R indexes [I>=2σ (I)] | R_1_ = 0.0555, wR_2_ = 0.1539 |
| Final R indexes [all data] | R_1_ = 0.0625, wR_2_ = 0.1596 |
| Largest diff. peak/hole / e Å^-3^ | 0.31/-0.26 |
| Flack parameter | -0.4(3) |

***Z*-α-(4-methoxyphenyl)-2’,3,4,4'-tetramethoxychalcone (*Z*-α-*p*-OMe-C_6_H_4_-TMC)**

CCDC no.: 1413000

*Crystal data and structure refinement*

| Empirical formula | C_26_H_26_O_6_ |
| --- | --- |
| Formula weight | 434.47 |
| Temperature/K | 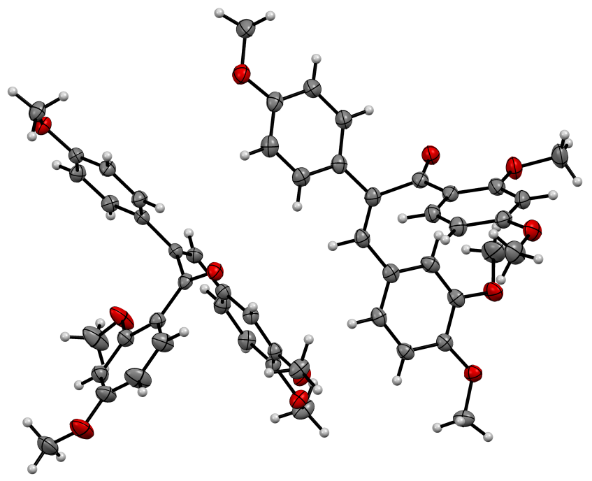123.01(10) |
| Crystal system | triclinic |
| Space group | P-1 |
| a/Å | 12.2050(6) |
| b/Å | 13.9442(5) |
| c/Å | 14.1638(6) |
| α/° | 77.245(3) |
| β/° | 69.721(4) |
| γ/° | 88.906(3) |
| Volume/Å^3^ | 2200.96(17) |
| Z | 4 |
| ρ_calc_g/cm^3^ | 1.311 |
| μ/mm^‑1^ | 0.759 |
| F(000) | 920.0 |
| Crystal size/mm^3^ | 0.208 × 0.116 × 0.07 |
| Radiation | CuKα (λ = 1.54184) |
| 2Θ range for data collection/° | 6.834 to 147.202 |
| Index ranges | -15 ≤ h ≤ 13, -17 ≤ k ≤ 17, -17 ≤ l ≤ 17 |
| Reflections collected | 30509 |
| Independent reflections | 8689 [R_int_ = 0.0334, R_sigma_ = 0.0261] |
| Data/restraints/parameters | 8689/0/587 |
| Goodness-of-fit on F^2^ | 1.031 |
| Final R indexes [I>=2σ (I)] | R_1_ = 0.0415, wR_2_ = 0.1064 |
| Final R indexes [all data] | R_1_ = 0.0509, wR_2_ = 0.1137 |
| Largest diff. peak/hole / e Å^-3^ | 0.26/-0.24 |

Supporting Table S1: Parameters of kinetic measurements

**Table S1** Wavelengths, fold thiol and time intervals (Δt) used in the kinetic assay.

| Compound | Wavelength / nm | [Compound]  / µM | Fold thiol*^a^* | Δt / s |
| --- | --- | --- | --- | --- |
| ***Z*-α-*p*-OMe-C_6_H_4_-TMC** | 315 | 40 | 1000-5000 | 1020 |

Fold thiol: **1000-5000:** 1000, 2000, 3000, 4000, 5000. *^a^* Cysteamine

^1^H NMR spectrum of *Z*-α-*p*-OMe-C_6_H_4_-TMC in CDCl_3_ at 400 MHz

^13^C NMR spectrum of *Z*-α-*p*-OMe-C_6_H_4_-TMC in CDCl_3_ at 101 MHz
